# Higher Physical Activity Is Associated with Improved Ventricular–Arterial Coupling: Assessment Using the cfPWV/GLS Ratio in Primary Care—A Pilot Study

**DOI:** 10.3390/jcdd12060208

**Published:** 2025-05-30

**Authors:** Paula-Anca Sulea, Ioan Tilea, Florin Stoica, Liviu Cristescu, Diana-Andreea Moldovan, Radu Tatar, Raluca-Maria Tilinca, Razvan Gheorghita Mares, Andreea Varga

**Affiliations:** 1 Doctoral School, George Emil Palade University of Medicine, Pharmacy, Science and Technology of Targu Mures, 540142 Targu Mures, Romania; paula-anca.almasan@umfst.ro (P.-A.S.); stoica.florin.23@stud.umfst.ro (F.S.); liviu.cristescu@umfst.ro (L.C.); diana.moldovan@umfst.ro (D.-A.M.); radu.tatar@umfst.ro (R.T.); 2 Faculty of Medicine, George Emil Palade University of Medicine, Pharmacy, Science and Technology of Targu Mures, 540142 Targu Mures, Romania; razvan.mares@umfst.ro; 3 Faculty of Medicine in English, George Emil Palade University of Medicine, Pharmacy, Science and Technology of Targu Mures, 540142 Targu Mures, Romania; andreea.varga@umfst.ro; 4 Mures County Clinical Hospital, 540072 Targu Mures, Romania; raluca.tilinca@gmail.com

**Keywords:** cardiovascular risk, vascular stiffening, metabolic equivalents, ventricular–arterial coupling, carotid–femoral pulse wave velocity, global longitudinal strain

## Abstract

Background: Age-related vascular stiffening increases cardiovascular risk by altering ventricular–arterial coupling (VAC). Physical activity, a modifiable factor, may improve cardiovascular health. This pilot study evaluated the relationship between physical activity evaluation and VAC, measured by the carotid–femoral pulse wave velocity to global longitudinal strain (cfPWV/GLS) ratio, in a Romanian primary care cohort. Methods: The prospective cohort analysis was performed on 81 adults (49 females, mean age 50.27 ± 12.93 years). Physical activity was quantified through anamnesis using metabolic equivalents (METs) according with Compendium of Physical Activities, and patients were stratified into four groups: G1 (METs < 1.5, *n* = 39), G2 (METs = 1.5–2.9, *n* = 2), G3 (METs = 3–5.9, *n* = 23), and G4 (METs ≥ 6, *n* = 17). Demographic and echocardiographic data were recorded to explore associations between physical activity and VAC. Results: The cfPWV/GLS ratio differed significantly across groups (*p* = 0.012), with the lowest values present in the moderate-intensity group (G3). VAC ≥ 0.391 can predict sedentary lifestyles (AUC = 0.730; CI: 0.617–0.833, *p* > 0.001). Multivariate analysis revealed that age, arterial age, and hypertension independently predict VAC. Conclusions: Higher physical activity is inversely associated with VAC (cfPWV/GLS ratio) and can predict sedentary lifestyles. Encouraging moderate-to-vigorous exercise in primary care may improve cardiovascular function and aid prevention.

## 1. Introduction

Aging and atherosclerosis are the principal contributors to increased arterial stiffness (AS), a central determinant in the pathogenesis of cardiovascular disease (CVD) [1,2]. Chronic inflammation, acting synergistically with dyslipidaemia, further accelerates atherosclerotic progression [3].

Hypertension (HTN), often preceding overt atherosclerosis, is a pivotal modifiable risk factor in cardiovascular prevention. Age-related arterial remodelling involves structural and functional alterations, predominantly characterized by wall stiffening, driven by mechanical stress and persistent inflammatory signalling that alter gene expression and remodel the extracellular matrix [4]. In large arteries, increased stiffness amplifies pulse pressures, primarily due to enhanced wave reflections [5].

Increased AS notably accelerates pulse wave velocity (PWV) [6]. Given its independent predictive value beyond traditional cardiovascular risk factors, assessing AS is clinically significant for risk stratification and therapeutic guidance. AS impacts myocardial function by elevating cardiac workload and impairing coronary perfusion, providing a mechanistic link between AS, HTN, left ventricular hypertrophy (LVH), and subsequent heart failure (HF) progression [7]. AS, a well-validated marker of vascular aging, is significantly higher in both habitual smokers and patients with diabetes mellitus (DM) than in age-matched healthy controls—equivalent to an advance in vascular age of several decades [8,9,10]. AS and wave reflections are independent predictors of adverse cardiovascular outcomes. As a hallmark of early vascular aging, AS represents a modifiable therapeutic target for preventing age-related organ damage and cardiovascular complications. By increasing cardiac workload and impairing coronary perfusion, AS contributes to LVH and progression to HF [11,12].

PWV is a widely accepted surrogate for AS and increases proportionally with vascular stiffening. Each 1 m/s rise in PWV is associated with a 12–14% increase in cardiovascular mortality [13]. Technological advances have expanded PWV assessment in primary care settings, enabling broader and more accessible evaluation [14,15].

AS is assessed using several parameters, including brachial–ankle PWV (baPWV), brachial–femoral PWV (bfPWV), and carotid–femoral PWV (cfPWV), the latter being the non-invasive gold standard. cfPWV is extensively used to evaluate arterial damage, stratify cardiovascular risk, and guide therapeutic decisions [16,17]. Its prognostic value has been validated across both general and high-risk populations, including hypertensive, diabetic and chronic kidney disease patients [18].

Recently developed devices combine cuff-based and tonometric techniques to enhance measurement accuracy [19]. cfPWV can also be measured alongside the ankle–brachial index (ABI), a validated marker of peripheral artery disease (PAD) and a strong predictor of coronary artery disease (CAD), particularly in men. However, in individuals with diabetes, ABI values may be falsely elevated due to medial arterial calcification, potentially obscuring underlying PAD [20,21,22].

Numerous studies support aerobic, isometric, and combined exercise as effective interventions for improving PWV in adults with HTN [23]. Cardiorespiratory fitness (CRF), quantified in metabolic equivalents (METs), provides a practical framework for prescribing safe and effective physical activity. Moderate-to-vigorous physical activity (≥3 METs) has been linked to reduced cardiovascular risk [24]. Low CRF levels are consistently associated with increased CVD incidence and all-cause mortality [25].

Global longitudinal strain (GLS) is a sensitive quantitative marker of subclinical systolic dysfunction, which often manifests in the basal segment of the interventricular septum in response to increased pressure overload and is particularly pronounced in hypertensive patients both with and without evidence of target-organ damage [26,27,28,29,30].

Ventricular–arterial coupling (VAC), which reflects the dynamic interplay between left ventricular contractility and arterial load (compliance, inertance and microvascular resistance), serves as an integrated marker of overall cardiovascular performance. VAC provides independent diagnostic and prognostic information, thereby enhancing risk stratification and guiding therapeutic monitoring. Optimal VAC ensures efficient stroke-volume delivery with stable blood pressure and preserved cardiovascular reserve [31,32,33]. Age-related vascular stiffening prompts compensatory LV remodelling at rest, preserving VAC but reducing reserve under stress—a process further influenced by comorbidities like HTN, diabetes, and chronic kidney disease. VAC parameters offer incremental predictive value beyond traditional risk factors [34,35].

The assessment of VAC has shown an independent prognostic value in different CVD. Chen et al. proposed that VAC can be quantified by the ratio of the effective arterial elastance (E_a_) to the end-systolic elastance (E_es_) of the left ventricle: VAC = $\frac{E_{a}}{E_{es}}$ [36].

Recent evidence positions the PWV/GLS ratio as a promising and comprehensive marker of VAC, offering notable advantages over the conventional echocardiographic E_a_/E_es_ ratio [37]. By combining PWV, an indicator for AS, with GLS, a sensitive indicator of myocardial function, this ratio provides an integrative assessment of cardiovascular interaction. Elevated PWV/GLS values are associated with ageing and increased cardiovascular risk, including HTN, obesity, and dyslipidaemia. As an emerging clinical tool, the PWV/GLS ratio may enhance early diagnosis, improve risk stratification, and support more personalised therapeutic decision making.

The objective of the current pilot study was to evaluate the effect of regular physical activity on VAC, as assessed by the carotid–femoral pulse wave velocity/global longitudinal strain (cfPWV/GLS) ratio, in a cohort of Romanian primary care patients.

## 2. Materials and Methods

### 2.1. Study Design and Population

This prospective pilot study enrolled 115 patients from 1 February 2023 to 1 April 2023 selected from an urban primary care office in central Romania.

Inclusion criteria encompassed adult female and male patients, Caucasian ethnicity. Exclusion criteria included a history of HF, valvular heart disease, CAD, pulmonary hypertension, chronic kidney disease, active infections, pregnancy, systemic inflammatory disorders, malignancies, cognitive impairment, or incomplete data availability or lost follow-up. Written informed consent was obtained from all participants prior to enrolment. Application of the predefined inclusion and exclusion criteria yielded a final study cohort of 81 eligible patients.

Data collected included demographic characteristics, comorbidities (obesity, HTN, and DM), smoking status, cfPWV parameters, and echocardiographic measurements.

Weekly physical exercise was evaluated using METs based on energy expenditure as described in the Compendium of Physical Activities [38]. Comprehensive patient anamnesis enabled quantification of both peak physical activity levels and habitual exercise patterns, which were subsequently expressed as standardized metabolic equivalent of task (MET) values. Based on these MET values, subjects were classified into four groups: group 1 (sedentary, G1, 1.0–1.5 METs), group 2 (light-intensity, G2, 1.6–2.9 METs), group 3 (moderate-intensity, G3, 3.0–5.9 METs), and group 4 (vigorous-intensity, G4, ≥6 METs) [39].

### 2.2. Clinical Assessment and Aortic Stiffness

Clinical data were extracted from Pharmec^©^ Healthcare Software platform version 6.1.574 (Cegedim RX/Pharmec Healthcare Software, Bucharest, Romania). Clinical assessments included measurements of abdominal circumference, body mass index (BMI), body surface area (BSA), and systolic and diastolic blood pressure (SBP, DBP). BMI and BSA were calculated using the Mosteller formula. BP and cfPWV were measured using a multicuff oscillometric diagnostic device—the MESI mTABLET Wireless Medical Tablet System (MESI, Ltd., Ljubljana, Slovenia). This user-tailored, non-invasive, and operator-independent device incorporates modules for resting ECG, pulse oximetry (SpO_2_), one-minute wireless ABI measurement, and cfPWV calculation. The oscillometric ABI value was attained from simultaneous BP measurements of all four extremities, performed in accordance with current recommendations [40,41].

### 2.3. Echocardiographic Parameters

A comprehensive transthoracic echocardiographic evaluation was performed using a GE Vivid^TM^ E9 System equipped with an M5Sc-D XDClear^TM^ probe (General Electric Vingmed Ultrasound AS, Horten, Norway) by a single experienced operator. Structural and functional cardiac parameters were acquired in accordance with the guidelines of the American Society of Echocardiography [42]. All acquired digital image loops were stored locally for offline analysis and subsequently reviewed by a second operator to ensure accuracy of measurements and interobserver reliability. Left ventricular ejection fraction (LVEF), left ventricular end-systolic volume (LVESV), stroke volume (SV) and cardiac output (CO) were calculated using Automated Ejection Fraction (Auto EF 2.0) measurement tool. GLS was evaluated as a key indicator of myocardial contractility via Automated Function Imaging (AFI 3.0) software tool.

### 2.4. Ventricular–Arterial Coupling (VAC) Assessment

VAC was evaluated using two distinct methodologies. The first approach involved calculating the ratio of effective arterial elastance (E_a_) to left ventricular end-systolic elastance (E_es_). E_a_ expresses all the extracardiac forces opposing ventricular ejection or arterial load and is commonly estimated using the formula E_a_ $=\frac{P_{ES}}{SV},$ where *P_ES_* denotes the end-systolic pressure (approximated by SBP), and SV represents stroke volume. E_es_ reflects cardiac contractility and is calculated using the formula E_es_ = $\frac{P_{ES}}{V_{ES}-V_{0}}$, where *V_ES_* is the end-systolic volume, and *V*_0_ is the theoretical ventricular volume at zero pressure.

The second method calculated VAC by deriving the ratio of cfPWV to the average GLS. These two indices were subsequently compared to evaluate the relationship between ventricular function and arterial properties across the patient groups.

### 2.5. Statistical Analysis

Statistical analyses were conducted using RStudio software version 4.2.3 (RStudio: Integrated Development for R. RStudio, PBC, Boston, MA, USA, http://www.rstudio.com/).

Normality of continuous numerical variables was assessed using the Kolmogorov–Smirnov test, and a significance threshold of α = 0.05 was applied for all tests. Continuous parametric variables were presented as mean ± standard deviation, while non-parametric variables were reported as median with interquartile ranges (IQR25–IQR75). Comparisons of central tendencies in unpaired parametric data were made using the independent samples *t*-test with or without equal variance assumptions based on the results of the variance test. The Mann–Whitney U test was used to compare central tendencies in unpaired non-parametric data. Categorical variables were analysed using the Chi-square test or Fisher’s exact test.

To examine the bivariate relationships between physical activity and VAC, both Spearman’s rank correlation coefficient and Pearson’s correlation coefficient were employed.

Linear regression models were constructed to quantify the association between physical activity levels and VAC. Additionally, logistic regression models were employed to assess the probability of abnormal VAC based on physical activity, adjusting for relevant covariates and potential confounders.

For comparisons among multiple groups, the Kruskal–Wallis test was used to evaluate differences in VAC across various physical activity levels. When significant differences were detected, post hoc pairwise comparisons were performed using the Dunn’s test to identify specific intergroup variations.

Scatter plots were used to examine correlations, and box plots facilitated group comparisons to explore variable distributions and relationships. In addition, receiver operating characteristics (ROC) curves with their corresponding area under the curve (AUC) values were applied to evaluate the classification model’s performance.

## 3. Results

The analysed cohort comprised 81 patients (mean age 50.3 ± 12.9 years) with preserved left ventricular ejection fraction (LVEF ≥ 50%). Of these, 49 (60.49%) were female, and 57 (70.37%) resided in urban areas. Cardiovascular risk factors included active smoking in 19 patients (23.5%), hypertension in 38 (46.9%), and DM in 6 (7.4%).

Of the 81 patients, 39 (48.1%) were classified as sedentary (G1). The remaining 42 (51.9%) were deemed physically active—2 (2.5%) in G2, 23 (28.4%) in G3, and 17 (21.0%) in G4—enabling a nuanced evaluation of activity levels. Baseline characteristics for the overall cohort, the sedentary subgroup, and the physically active subgroup are presented in Table 1 and Table 2, respectively.

Comparison of comprehensive cardiac performance and AS metrics among all four groups is provided in Table 3.

**Figure 1:**
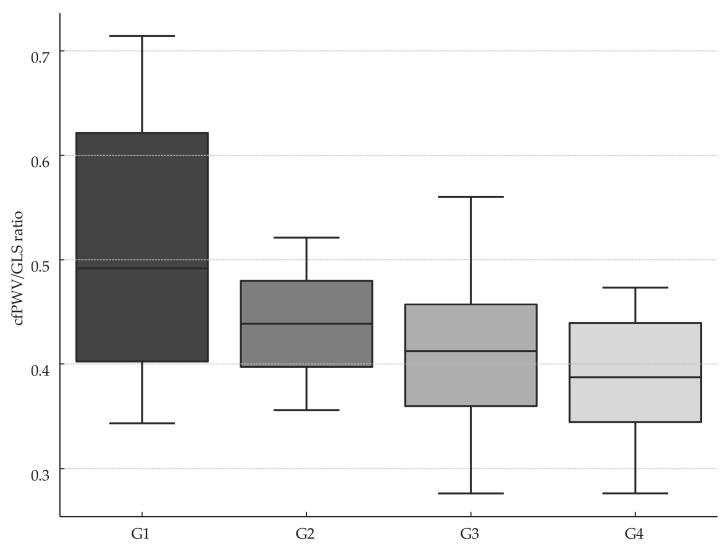
Box plot of VAC across patient groups stratified according to METs. cfPWV, carotid–femoral pulse wave velocity; GLS, global longitudinal strain; G1, group 1; G2, group 2; G3, group 3; G4, group 4; VAC, ventricular–arterial coupling, expressed as the ratio between cfPWV and average GLS.

In the overall cohort, 42 of 81 patients had METs ≥ 1.5. Analysis of the cfPWV/GLS ratio demonstrated significant differences among the four activity groups (*p* = 0.012), with the lowest mean cfPWV/GLS observed in the vigorous-intensity group (G4) (Figure 1).

Post hoc analysis using Dunn’s test demonstrated that the sedentary group (G1) differed significantly from both the moderate-intensity group (G3) and the vigorous-intensity group (G4) (*p* < 0.05 for each), with no other pairwise contrasts reaching statistical significance (Table 4).

A moderate, statistically significant negative correlation was found between VAC (cfPWV/GLS) and LVEF in physically active patients (r = −0.55, *p* < 0.001).

Additionally, a statistically significant positive correlation was observed between BMI and VAC (cfPWV/GLS ratio, r = 0.31, *p* = 0.018).

Pearson analysis showed a moderate, significant positive correlation between DBP and VAC (cfPWV/GLS ratio, r = 0.48, *p* < 0.001) and between SBP and VAC (cfPWV/GLS ratio, r = 0.36, *p* = 0.006).

Linear regression showed a significant inverse relationship between METs and the VAC (cfPWV/GLS), with each one-unit METs increase linked to a 0.014-unit decrease (r^2^ = 0.10, *p* = 0.005). cfPWV/GLS ratio was inversely associated with LVEF (β = −18.63, r^2^ = 0.25, *p* < 0.001) and METs (β = −7.17, r^2^ = 0.10, *p* < 0.005), underscoring its link with both cardiac function and physical activity.

A multivariate linear regression model was developed to evaluate the associations between selected parameters and VAC quantified by the cfPWV/GLS ratio. The model specification was as follows (Table 5):

*Y* = *β*_0_ + *β*_1_(*female*) + *β*_2_(*right ABI*) + *β*_3_(*left ABI*) + *β*_4_(*DBP*) + *β*_5_(*arterial age*) + *β*_6_(*SpO*_2_) + *β*_7_(*HTN*) + *β*_8_(*DM*) + *β*_9_(*BMI*) + *β*_10_(*LVPW*) + *β*_11_(*CO*)

The multivariate linear regression model explained 81% of the variance in ventricular–arterial coupling (cfPWV/GLS ratio). Independent predictors significantly associated with VAC included right ABI, left ABI, arterial age, SpO_2_, hypertension, presence of DM, left ventricular posterior wall dimensions, and cardiac output.

Spearman’s correlation analysis was performed to evaluate associations between VAC and multiple parameters (Table 6). Age showed a stronger correlation with VAC when measured by the cfPWV/GLS ratio than by the E_a_/E_es_ ratio. Additionally, body surface area (BSA) reached statistical significance only in the cfPWV/GLS model, suggesting that this method may provide a more sensitive assessment of VAC than the traditional E_a_/E_es_ approach.

### 3.1. Comparative Analysis Between VAC Derived from E_a_/E_es_ and the cfPWV/GLS Ratio

The predictive value of the E_a_/E_es_ and cfPWV/GLS ratios in identifying sedentary individuals was evaluated using ROC curve analysis, with sedentary behaviour as the dependent variable and VAC as the independent variable.

When VAC was calculated as E_a_/E_es_ ratio, an optimal cut-off of 0.796 was determined for predicting sedentarism. At this threshold, sensitivity was 64.5%, and specificity was 76.9%, according to the maximum Youden’s index. The AUC was approximately 0.690 (95% CI: 0.560–0.820) with a statistically significant *p*-value (*p* < 0.001), indicating sufficient discriminatory capacity.

In contrast, when VAC was estimated using the cfPWV/GLS ratio, the optimal cut-off value for predicting sedentarism was 0.391. At this threshold, sensitivity was 90.3% and the specificity 46.2%. The corresponding area under the curve was 0.730 (95% CI: 0.617–0.833; *p* < 0.001), indicating good discriminatory performance.

**Figure 2:**
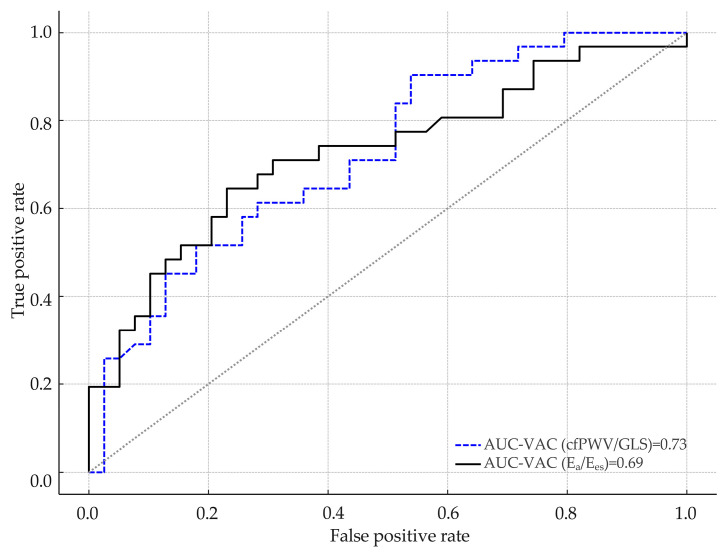
Comparison of ROC curves for identifying sedentary individuals. AUC, area under the curve; cfPWV, carotid–femoral pulse wave velocity; E_a_, effective arterial elastance; E_es_, left ventricular end-systolic elastance; GLS, global longitudinal strain; VAC, ventricular–arterial coupling.

ROC curve comparison did not emphasize any differences between cfPWV/GLS ratio and E_a_/E_es_ for predicting sedentary individuals (*p* = 0.657, DeLong test). Data are presented in Figure 2.

Logistic regression showed that female gender was associated with lower cfPWV values (OR: 0.72, 95% CI: 0.51–0.98, *p* = 0.040), while a sedentary lifestyle increased the risk of elevated cfPWV (OR: 1.67, 95% CI: 1.02–2.46, *p* = 0.004).

A separate multivariate logistic regression model (r^2^ = 0.221, *p* = 0.036) for predicting VAC ≥ 0.391—the threshold derived from ROC curve analysis—is presented in Table 7. Adherence to antihypertensive and antidiabetic therapies was independently associated with lower odds of elevated VAC values.

These findings indicate that the cfPWV/GLS ratio may serve as a useful marker in identifying individuals at risk of sedentarism, although further validation in larger cohorts is warranted.

### 3.2. Comparative Assessment Between VAC and Echocardiographic Parameters

To evaluate the hypothesis that the echocardiographic parameters of myocardial function and structure could influence VAC, a Spearman correlation analysis was performed. The results revealed significant differences in correlation strength between the two methods, with the cfPWV/GLS ratio demonstrating notably stronger associations (Table 8).

## 4. Discussion

Our findings are consistent with prior studies showing that increased physical activity reduces AS and VAC, highlighting physical activity as a key modifiable factor in cardiovascular risk reduction. AS is clinically relevant not only for its impact on cardiac function but also for its role in vascular wall dysfunction and atherogenesis [43].

Statistical analyses showed a moderate inverse correlation between cfPWV and METs, as well as a significant association with female gender. Sedentary behaviour emerged as a risk factor for elevated cfPWV, aligning with evidence from Bohn et al., who reported an independent link between sedentary lifestyle and increased cfPWV (*p* = 0.010) [44]. Similarly, a large European multicenter study identified age and hypertension as independent determinants of AS, measured by both cardio–ankle vascular index (CAVI) and cfPWV [45].

These findings highlight the critical role of early lifestyle interventions—particularly structured exercise—in delaying or reversing subclinical cardiovascular abnormalities in patients with metabolic syndrome, potentially reducing morbidity and mortality [46].

In a landmark study, Lee et al. estimated that physical inactivity contributes to nearly 9% of premature deaths globally, with its elimination projected to raise global life expectancy by 0.68 years [47]. Current guidelines recommend 500–1000 MET minutes of aerobic activity per week, which is equivalent to 150–300 min of moderate or 75–150 min of vigorous-intensity exercise [48].

A comprehensive systematic review and meta-analysis by Abell et al. demonstrated that exercise-based cardiac rehabilitation significantly improves clinical outcomes in patients with CAD, including reductions in cardiovascular and all-cause mortality, as well as myocardial infarction incidence. In addition to its prognostic benefits, rehabilitation enhances functional capacity, alleviates symptoms, and improves quality of life, particularly in patients with advanced or symptomatic disease [49].

Patients at risk of developing HTN may benefit from early evaluation of AS, as emerging evidence suggests that increased central AS—typically measured by cfPWV—often precedes the clinical onset of hypertension. Elevated AS facilitates the transmission of pulsatile pressure and flow to microvascular beds, thereby contributing to end-organ damage. A recent meta-analysis has established that AS is independently associated with adverse cardiovascular outcomes, including CAD and stroke. These findings underscore the prognostic relevance of AS as a subclinical marker and reinforce its potential role in early risk stratification and prevention strategies [50].

Jeong et al. reported that individuals with pre-existing CVD tend to adopt more sedentary lifestyles, despite their greater potential to benefit from regular physical activity. Physical activity levels exceeding 500–1000 MET minutes per week are associated with significant clinical benefits [51].

Contemporary guidelines endorse the routine incorporation of structured exercise training into the management of all patients with CVD—especially those with CAD and HF—and recommend referral to individualized cardiac rehabilitation and supervised exercise training programs [52,53].

In a prior study, the PWV/GLS ratio demonstrated significant associations with adverse cardiovascular risk factors independent of age, suggesting it may offer superior utility compared with E_a_/E_es_ ratio for assessing VAC [54].

The PWV/GLS ratio has demonstrated clinical value in identifying impaired VAC in patients with HTN. In a comparative study, both hypertensive individuals and those with coexisting CAD exhibited significantly reduced GLS and alongside elevated PWV values relative to healthy controls. Consequently, VAC—when assessed via the PWV/GLS ratio—was markedly disrupted in these groups, underscoring its value for early detection of subclinical cardiovascular dysfunction [55].

Our findings showed that VAC, expressed as the cfPWV/GLS ratio, varied significantly across physical activity subgroups—sedentary, light-, moderate-, and vigorous-intensity—with the lowest values observed in the moderate-intensity group (METs 3–5.9). These data concur with recent evidence showing that structured exercise interventions enhance GLS—and thereby lower VAC—in patients with CVD, underscoring GLS’s promise as an early biomarker of exercise-induced improvements in ventricular–arterial coupling [56].

Furthermore, a prospective cohort study reinforced the existence of a threshold of moderate-to-vigorous physical activity that confers maximal protection against arterial stiffness. The greatest benefit was observed in individuals performing exercise equal to 5.50–9.70 MET hours per day [57].

In the STANISLAS cohort, VAC derived from the conventional E_a_/E_es_ formula displayed an inverse relationship with chronological age—an unexpected finding that contradicts established physiological patterns and calls into question the validity of the formula-based approach. By contrast, VAC quantified via the PWV/GLS ratio has been shown to correlate positively with age, a trend that our data also confirm [58]. In our study, older participants exhibited higher PWV/GLS-derived VAC values, further substantiating the progressive, age-related deterioration of ventricular–arterial coupling.

In symptomatic patients, PWV has been associated with both the presence and severity of CAD as assessed by invasive coronary angiography (ICA). One study demonstrated a strong positive correlation between CAD severity and cfPWV in 103 patients undergoing ICA [58]. Similarly, Hofmann et al. reported a significant association between elevated cfPWV and both the presence and severity of CAD in a cohort of 155 patients [59]. Elevated PWV has also been implicated in the development of large cerebral artery calcification, luminal stenosis, and occlusion, findings documented in both hypertensive cohorts and patients presenting with acute ischemic stroke [60].

Despite its proven utility for early detection and risk stratification in cardiovascular disease, PWV has seen limited applicability in routine clinical practice, largely owing to methodological constraints and variable interpretability. PWV values are modulated by several patient-related factors—most notably blood pressure, but also age and smoking status—with acute changes in BP representing the principal source of measurement variability. Increased BP augments the arterial wall tension and adds functional AS. Exercise, smoking cessation, and medication can reduce AS and decrease PWV value [61].

The MESI mTABLET system enabled rapid, non-invasive acquisition of cfPWV and ABI, demonstrating the feasibility of integrating VAC assessment into routine primary care.

### 4.1. Study Limitations

Several limitations warrant consideration. The modest sample size and single-center design may restrict external validity, particularly in non-urban or demographically diverse settings. As an observational, cross-sectional study, causal inferences between habitual physical activity and ventricular–arterial coupling cannot be drawn. Measurements of cfPWV and GLS were obtained on a single device platform, which may limit comparability with other techniques such as applanation tonometry or alternative cfPWV assessment tools [62]. Additionally, some patients were lost to follow-up, contributing to potential selection bias. Lastly, physical activity was assessed retrospectively through patient-reported anamnesis, which may introduce recall bias and reduce the precision of METs estimation.

### 4.2. Future Directions

Given these limitations, a prospective longitudinal study assessing the reproducibility and evolution of cfPWV/GLS values over time in relation to sustained physical activity would be of significant value. Repeated measurements could clarify the trajectory of VAC and help determine the long-term impact of lifestyle interventions in primary care populations. Conducting randomized controlled trials that compare structured exercise interventions to standard of care would offer stronger evidence for the therapeutic value of physical activity in improving VAC and reducing cardiovascular risk, especially in primary care settings.

Integrating circulating biomarkers such as NT-proBNP, hs-CRP, or other proposed biomarkers could provide additional insight into the biological pathways linking physical activity to myocardial and vascular function to a broader spectrum of pathologies [63,64]. Evaluating how cfPWV/GLS can be embedded into cardiovascular risk assessment models or electronic decision support systems could promote its adoption as a screening or monitoring tool in general practice. Future research could also explore how antihypertensive, lipid-lowering, or antidiabetic therapies influence cfPWV/GLS over time, particularly when combined with lifestyle interventions.

## 5. Conclusions

This pilot study demonstrates that higher physical activity levels—particularly moderate-intensity exercise—are significantly associated with improved VAC, as measured by the cfPWV/GLS ratio. Compared to the traditional E_a_/E_es_ method, the cfPWV/GLS ratio showed stronger associations with cardiovascular and echocardiographic parameters and higher discriminatory ability in identifying sedentary individuals.

These findings support the use of VAC derived from the cfPWV/GLS ratio as a sensitive, practical tool for cardiovascular risk stratification in primary care. Further studies are needed to validate and extend these results.

## Figures and Tables

**Table 1:** Baseline characteristics of the sedentary and physically active patients.

Characteristics	Total (*n* = 81)	Sedentary (*n* = 39)	Physical Active (*n* = 42)	*p* Value
Age, years (mean ± SD)	50.27 ± 12.93	57.46 ± 11.83	43.60 ± 10.09	<0.001
Female (*n*, %)	49 (60.5)	26 (66.67)	23 (54.76)	ns
Urban (*n*, %)	57 (70.37)	26 (66.67)	31 (73.81)	ns
Baseline risk factors				
Abdominal circumference, cm (mean ± SD)	101.50 ± 14.43	106.30 ± 14.48	94.12 ± 10.89	<0.001
BMI, kg/m^2^ (mean ± SD)	28.41 ± 5.78	30.19 ± 6.09	25.56 ± 3.82	<0.001
BSA, m^2^ (mean ± SD)	1.92 ± 0.24	1.95 ± 0.24	1.88 ± 0.24	ns
HTN (mmHg, %)	38 (46.91)	26 (66.67)	12 (28.57)	0.001
DM (*n*, %)	6 (7.41)	6 (15.38)	0 (0)	0.030
Active smoking (*n*, %)	19 (23.46)	8 (20.51)	11 (26.19)	ns
Cardiovascular parameters				
SBP, mmHg (mean ± SD)	124.36 ± 15.66	128.69 ± 16.77	120.98 ± 14.02	0.036
DBP, mmHg (mean ± SD)	76.57 ± 9.77	76.60 ± 9.42	76.51 ± 10.40	ns
SpO_2_ (mean ± SD)	0.97 ± 0.02	0.96 ± 0.03	0.98 ± 0.01	<0.001
cfPWV, m/s (mean ± SD)	8.75 ± 1.70	9.30 ± 1.82	8.13 ± 1.31	0.005
LVPW, mm (median, IQR)	9.0 (8.0–10.0)	9.0 (9.0–10.0)	9.0 (8.0–10.0)	ns
LVESV, mL (mean ± SD)	41.76 ± 13.60	42.92 ± 14.45	40.85 ± 13.01	ns
SV, mL (mean ± SD)	54.90 ± 15.80	51.61 ± 14.42	57.46 ± 16.52	ns
CO, L/min (median, IQR)	3.7 (3.1–4.4)	3.6 (2.8–4.4)	3.9 (3.3–4.5)	ns
E_a_ (median, IQR)	2.24 (1.91–2.85)	2.71 (2.04–3.13)	2.17 (1.85–2.49)	0.013
E_es_ (median, IQR)	3.10 (2.52–3.93)	3.22 (2.44–4.00)	3.08 (2.53–3.83)	ns
GLS A3C (mean ± SD)	18.92 ± 3.18	18.19 ± 3.37	19.45 ± 2.95	ns
GLS A4C (mean ± SD)	18.11 ± 2.98	17.61 ± 3.25	18.47 ± 2.74	ns
GLS A2C (mean ± SD)	19.77 ± 3.18	18.13 ± 2.98	21.01 ± 2.76	<0.001
GLS, % (mean ± SD)	17.52 ± 4.75	15.63 ± 5.50	19.54 ± 2.59	<0.001
$\mathrm{VAC}\;(\frac{E_{a}}{E_{es}})$ (mean ± SD)	0.77 ± 0.20	0.85 ± 0.24	0.72 ± 0.13	0.007
VAC (cfPWV/GLS ratio) (median, IQR)	0.43 (0.38–0.53)	0.49 (0.40–0.62)	0.40 (0.35–0.45)	0.001

BMI, body mass index; BSA, body surface area; cfPWV, carotid–femoral pulse wave velocity; CO, cardiac output; DBP, diastolic blood pressure; DM, diabetes mellitus; E_a_, effective arterial elastance; E_es_, left ventricular end-systolic elastance; GLS, global longitudinal strain; GLS A2C, global longitudinal peak systolic strain apical two chambers; GLS A3C, global longitudinal peak apical three chamber; GLS A4C, apical global longitudinal peak systolic strain four chambers; HTN, hypertension; IQR, interquartile range; LVESV, left ventricular end-systolic volume; LVPW, left ventricular posterior wall; *n*, number of patients; ns, non-significant; SBP, systolic blood pressure; SD, standard deviation; SpO_2_, peripheral oxygen saturation; SV, stroke volume; VAC, ventricular–arterial coupling.

**Table 2:** Baseline characteristics of physical active patients.

Characteristics	Total Physical Active Patients (*n* = 42)	G2 (*n* = 2)	G3 (*n* = 23)	G4 (*n* = 17)	*p* Value
Age, years (mean ± SD)	43.60 ± 10.09	46.00 ± 25.46	45.26 ± 8.63	41.06 ± 10.32	ns
Female (*n*, %)	23 (54.76)	1 (50.00)	16 (69.57)	6 (35.29)	<0.001
Urban (*n*, %)	31 (73.81)	2 (100.00)	15 (65.22)	14 (82.35)	ns
Baseline risk factors					
Abdominal circumference, cm (mean ± SD)	93.73 ± 10.26	85.75 ± 6.72	93.85 ± 9.84	94.50 ± 11.20	ns
BMI, kg/m^2^ (mean ± SD)	25.43 ± 3.50	24.00 ± 2.12	24.68 ± 3.62	26.61 ± 3.25	ns
BSA, m^2^ (mean ± SD)	1.87 ± 0.24	1.80 ± 0.24	1.80 ± 0.21	1.98 ± 0.25	0.049
HTN (mmHg, %)	12 (28.57)	1 (50.00)	6 (26.09)	5 (29.41)	ns
Current smoking (*n*, %)	8 (19.05)	1 (50.00)	5 (21.74)	2 (11.76)	ns
Cardiovascular parameters					
SBP, mmHg (mean ± SD)	120.93 ± 13.86	136.00 ± 28.28	116.91 ± 13.02	124.59 ± 11.95	ns
DBP, mmHg (mean ± SD)	77.00 ± 9.90	80.00 ± 19.80	76.57 ± 10.26	77.24 ± 8.98	ns
SpO_2_ (mean ± SD)	0.98 ± 0.01	0.98 ± 0.02	0.98 ± 0.01	0.98 ± 0.01	ns
cfPWV, m/s (mean ± SD)	8.11 ± 1.29	8.00 ± 0.85	8.26 ± 1.41	7.93 ± 1.20	ns
LVPW, mm (mean ± SD)	9.14 ± 1.34	8.00 ± 1.41	8.96 ± 1.22	9.53 ± 1.42	ns
LVESV, mL (median, IQR)	40.85 ± 13.01	42.50 ± 19.09	36.61 ± 10.92	46.75 ± 13.68	ns
SV, mL (mean ± SD)	57.90 ± 16.49	61.50 ± 13.44	50.82 ± 13.80	67.19 ± 16.15	0.006
CO, L/min (median, IQR)	3.96 ± 1.09	4.10 ± 0.14	3.65 ± 1.01	4.38 ± 1.16	ns
E_a_ (mean ± SD)	2.25 ± 0.62	2.21 ± 0.02	2.45 ± 0.62	1.97 ± 0.58	0.05
E_es_ (mean ± SD)	3.21 ± 0.91	3.39 ± 0.86	3.42 ± 0.92	2.88 ± 0.87	ns
GLS A3C (mean ± SD)	19.45 ± 2.95	18.65 ± 4.31	19.32 ± 2.69	19.73 ± 3.32	ns
GLS A4C (mean ± SD)	18.47 ± 2.74	17.30 ± 2.83	18.67 ± 2.64	18.35 ± 2.99	ns
GLS A2C (mean ± SD)	21.01 ± 2.76	19.95 ± 2.19	21.05 ± 2.86	21.09 ± 2.81	ns
GLS, % (mean ± SD)	19.77 ± 2.40	18.65 ± 3.04	19.91 ± 2.23	19.73 ± 2.65	ns
$\mathrm{VAC}\;(\frac{E_{a}}{E_{es}})$ (mean ± SD)	0.72 ± 0.13	0.67 ± 0.16	0.74 ± 0.15	0.69 ± 0.10	ns
VAC (cfPWV/GLS ratio) (mean ± SD)	0.42 ± 0.10	0.44 ± 0.12	0.42 ± 0.10	0.41 ± 0.11	ns

BMI, body mass index; BSA, body surface area; cfPWV, carotid–femoral pulse wave velocity; CO, cardiac output; DBP, diastolic blood pressure; E_a_, effective arterial elastance; E_es_, left ventricular end-systolic elastance; GLS, global longitudinal strain; GLS A2C, global longitudinal peak systolic strain apical two chambers; GLS A3C, global longitudinal peak apical three chamber; GLS A4C, apical global longitudinal peak systolic strain four chambers; HTN, hypertension; IQR, interquartile range; LVESV, left ventricular end-systolic volume; LVPW, left ventricular posterior wall; *n*, number of patients; ns, non-significant; SBP, systolic blood pressure; SD, standard deviation; SpO_2_, peripheral oxygen saturation; SV, stroke volume; VAC, ventricular–arterial coupling.

**Table 3:** Cardiac performance and arterial stiffness metrics among all patient groups (G1–G4).

Parameter	G1 (*n* = 39)	G2 (*n* = 2)	G3 (*n* = 23)	G4 (*n* = 17)	*p* Value
GLS A3C (mean ± SD)	18.19 ± 3.37	18.65 ± 4.31	19.32 ± 2.69	19.73 ± 3.32	ns *
GLS A4C (mean ± SD)	17.61 ± 3.25	17.3 ± 2.83	18.67 ± 2.64	18.35 ± 2.99	ns *
GLS A2C (mean ± SD)	18.13 ± 2.98	19.95 ± 2.19	21.05 ± 2.86	21.09 ± 2.81	0.001 *
GLS average (mean ± SD)	17.99 ± 2.64	18.65 ± 3.04	19.91 ± 2.23	19.73 ± 2.65	0.035 *
LVEF, % (median, IQR)	55.00 (52.00–57.50)	60.00 (58.00–62.00)	59.50 (56.00–61.00)	58.00 (56.25–62.75)	0.002 **
cfPWV (m/s) (median, IQR)	9.00 (8.10–9.80)	8.00 (7.70–8.30)	7.90 (7.15–9.25)	7.65 (7.32–8.92)	0.021 **
$\mathrm{VAC}\;(\frac{E_{a}}{E_{es}})$, (median, IQR)	0.85 (0.69–0.94)	0.67 (0.62–0.73)	0.73 (0.64–0.80)	0.71 (0.62–0.79)	0.027 **
VAC (cfPWV/GLS ratio), (median, IQR)	0.49 (0.40–0.62)	0.43 (0.39–0.47)	0.40 (0.35–0.45)	0.39 (0.34–0.43)	0.013 **

cfPWV, carotid–femoral pulse wave velocity; E_a_, effective arterial elastance; E_es_, left ventricular end-systolic elastance; GLS, global longitudinal strain, GLS A2C, global longitudinal peak systolic strain apical two chambers; GLS A3C, global longitudinal peak apical three chamber; GLS A4C, global longitudinal peak systolic strain apical four chambers, IQR, interquartile range; LVEF, left ventricular ejection fraction; *n*, number of patients; SD, standard deviation, VAC, ventricular–arterial coupling; * = parametric ANOVA test; ** = non-parametric Kruskal–Wallis test.

**Table 4:** Post hoc analysis among the four groups.

Dunn’s Multiple Comparison Test	Mean Rank Difference	Adjusted *p* Value
G1 vs. G2	11.060	0.999
G1 vs. G3	15.520	0.046
G1 vs. G4	17.390	0.031
G2 vs. G3	4.452	>0.999
G2 vs. G4	6.324	>0.999
G3 vs. G4	1.871	>0.999

G1, group 1; G2, group 2; G3, group 3; G4, group 4.

**Table 5:** Ventricular–arterial coupling multivariate linear regression model results.

cfPWV/GLS Ratio	Estimated Coefficient	*p* Value
Intercept (*β*_0_)	0.809	ns
Sex (female) (*β*_1_)	−0.016	ns
Right ABI (*β*_2_)	−0.281	0.049
Left ABI (*β*_3_)	0.393	0.009
DBP (mmHg) (*β*_4_)	0.002	ns
Arterial age (years) (*β*_5_)	0.006	<0.001
SpO_2_ (*β*_6_)	−0.010	0.027
HTN (*β*_7_)	−0.052	0.003
DM (*β*_8_)	0.095	0.032
BMI (*β*_9_)	0.003	ns
LVPW (*β*_10_)	0.002	0.002
CO (*β*_11_)	−0.001	ns
r^2^	0.81	

ABI, ankle–brachial index; BMI, body mass index; CO, cardiac output; DBP, diastolic blood pressure; DM, diabetes mellitus; HTN, hypertension; LVPW, left ventricular posterior wall, SpO_2_, peripheral oxygen saturation.

**Table 6:** Correlation analysis between both VAC methods and evaluated parameters.

Parameters	VAC (E_a_/E_es_)		VAC (cfPWV/GLS)	
	r	*p* Value	r	*p* Value
Age	0.38	0.005	0.52	<0.001
Abdominal circumference	0.36	0.003	0.32	0.008
BMI	0.34	0.004	0.28	0.018
BSA	0.19	0.110	0.24	0.040
METs	−0.37	0.002	−0.34	0.004

BMI, body mass index; BSA, body surface area; cfPWV, carotid–femoral pulse wave velocity; E_a_, effective arterial elastance; E_es_, left ventricular end-systolic elastance; GLS, global longitudinal strain; METs, metabolic equivalents of task; VAC, ventricular–arterial coupling.

**Table 7:** Multivariate logistic regression for elevated VAC (cfPWV/GLS ≥ 0.391).

Variable	Coefficient	Standard Error	Odds Ratio	95% CI Lower	95% CI Upper	*p*-Value
Intercept	−50.673	25.953	0.000	0.000	1.215	0.051
Female sex	−1.573	0.762	0.207	0.047	0.922	0.039
Age	0.127	0.041	1.136	1.048	1.232	0.002
Smoker	0.277	0.714	1.319	0.325	5.345	0.699
Treated hypertension	−0.520	0.803	0.595	0.123	2.869	0.517
Treated DM	−0.243	1.351	0.784	0.052	11.769	0.857
BMI	0.140	0.122	1.150	0.904	1.462	0.250
SpO_2_	0.087	0.109	1.091	0.878	1.357	0.424

BMI, body mass index; SpO_2_, peripheral oxygen saturation.

**Table 8:** Correlations between echocardiographic parameters and VAC values for the entire cohort.

Parameters	VAC (E_a_/E_es_)		VAC (cfPWV/GLS)	
	r	*p* Value	r	*p* Value
LAESD	0.24	ns	0.19	ns
LAA	0.18	ns	0.07	ns
LAV	0.26	0.041	−0.02	ns
E/A ratio	−0.41	0.001	−0.61	<0.001
Maximum E velocity	−0.37	0.002	−0.44	<0.001
Maximum e’ velocity (septal)	−0.55	<0.001	−0.71	<0.001
E/e’ (septal)	0.33	0.008	0.39	0.002
Maximum e’ velocity (lateral)	−0.33	0.008	−0.49	<0.001
Lateral E/e’	0.07	ns	0.17	ns
Maximum e’ velocity (mean)	−0.46	<0.001	−0.61	<0.001

A, late (atrial) transmitral pulse wave Doppler flow; cfPWV, carotid–femoral pulse wave velocity; E, early transmitral pulse wave Doppler flow; e’, velocity of early myocardial relaxation measured on tissue Doppler imaging; E_a_, effective arterial elastance; E_es_, left ventricular end-systolic elastance; E/A, early to late diastolic transmitral flow velocity; GLS, global longitudinal strain; LAA, left atrial area; LAESD, left atrial end systolic diameter; LAV, left atrial volume; V, velocity; VAC, ventricular–arterial coupling.

## Data Availability

Additional information can be obtained from the corresponding author according to the local and national regulations.

## Abbreviations

The following abbreviations are used in this manuscript:

A2C	Apical two-chamber view
A3C	Apical three-chamber view
A4C	Apical four-chamber view
ABI	Ankle brachial index
AUC	Area under the curve
baPWV	Brachial–ankle pulse wave velocity
bfPWV	Brachial–femoral pulse wave velocity
BMI	Body mass index
BP	Blood pressure
BSA	Body surface area
CAD	Coronary artery disease
CAVI	Cardio–ankle vascular index
cfPWV	Carotid–femoral pulse wave velocity
CI	Confidence interval
CO	Cardiac output
CRF	Cardio-respiratory fitness
CVD	Cardiovascular disease
DBP	Diastolic blood pressure
DM	Diabetes mellitus
E_a_	Arterial elastance
E_es_	Left ventricular end-systolic elastance
eNOS	Endothelial nitric oxide synthase
GLS	Global longitudinal strain
HF	Heart failure
HFpEF	Heart failure with preserved ejection fraction
HTN	Hypertension
ICA	Invasive coronary angiography
IQR	Interquartile range
LAA	Left atrial area
LAESD	Left atrial end systolic diameter
LAV	Left atrial volume
LV	Left ventricle or left ventricular
LVEF	Left ventricular ejection fraction
LVESV	Left ventricular end-systolic volume
LVH	Left ventricular hypertrophy
LVPW	Left ventricular posterior wall
METs	Metabolic equivalents of task (plural)
NO	Nitric oxide
PAD	Peripheral artery disease
P_ES_	End-systolic pressure
PWV	Pulse wave velocity
ROC	Receiver operating characteristic
SBP	Systolic blood pressure
SD	Standard deviation
SpO_2_	Peripheral oxygen saturation
SV	Stroke volume
VAC	Ventricular–arterial coupling
V_ES_	End-systolic volume
